# The EXPANd trial: effects of exercise and exploring neuroplastic changes in people with Parkinson’s disease: a study protocol for a double-blinded randomized controlled trial

**DOI:** 10.1186/s12883-019-1520-2

**Published:** 2019-11-12

**Authors:** Erika Franzén, Hanna Johansson, Malin Freidle, Urban Ekman, Martin Benka Wallén, Ellika Schalling, Alexander Lebedev, Martin Lövdén, Staffan Holmin, Per Svenningsson, Maria Hagströmer

**Affiliations:** 1 Department of Neurobiology, Care sciences and Society, Division of Physiotherapy, Karolinska Institutet, Stockholm, Sweden; 2 Karolinska University Hospital, Allied Health Professionals Function, Function Area Occupational Therapy & Physiotherapy, Stockholm, Sweden; 3 Stockholms Sjukhem, R&D unit, Stockholm, Sweden; 4 Department of Neurobiology, Care sciences and Society, Division of Clinical Geriatrics, Karolinska Institutet, Stockholm, Sweden; 5 Karolinska University Hospital, Allied Health Professionals Function, Function Area Medical Psychology, Stockholm, Sweden; 6 Department of Clinical Science, Intervention and Technology, CLINTEC, Division of Speech and Language Pathology, Karolinska Institutet, Stockholm, Sweden; 7 Karolinska University Hospital, Allied Health Professionals Function, Function Area Speech and Language Pathology, Stockholm, Sweden; 8 Department of Neurobiology, Care sciences and Society, Aging Research Center, Karolinska Institutet, Stockholm, Sweden; 9 Department of Clinical Neuroscience, Division of Neurology, Karolinska Institutet, Stockholm, Sweden; 10 Department of Health Promoting Science, Sophiahemmet University, Stockholm, Sweden

**Keywords:** Balance, Cognition, Dual-task, Exercise, Gait, Imaging, Magnetic resonance neural growth factor, Neuroplasticity, Parkinson’s disease

## Abstract

**Background:**

Parkinson’s disease (PD) affects many physiological systems essential for balance control. Recent studies suggest that intensive and cognitively demanding physical exercise programs are capable of inducing plastic brain changes in PD. We have developed a highly challenging balance training (the HiBalance) program that emphasizes critical aspects of balance control through progressively introducing more challenging exercises which incorporates dual-tasking. Earlier studies have shown it to be effective in improving balance, gait and dual-tasking. The study design has thereafter been adjusted to link intervention-induced behavioral changes to brain morphology and function. Specifically, in this randomized controlled trial, we will determine the effects of the HiBalance program on balance, gait and cognition and relate this to task-evoked functional MRI (fMRI), as well as brain-derived neurotrophic factor (BDNF) in participants with mild-moderate PD.

**Methods:**

One hundred participants with idiopathic PD, Hoehn & Yahr stage 2 or 3, ≥ 60 years of age, ≥ 21 on Montreal Cognitive Assessment will be recruited in successive waves and randomized into either the HiBalance program or to an active control group (the HiCommunication program, targeting speech and communication). Both interventions will be performed in small groups, twice a week with 1 h sessions for 10 weeks. In addition, a 1 h, once a week, home exercise program will also be performed. A double-blinded design will be used. At the pre- and post-assessments, participants will be assessed on balance (main outcome), gait, cognitive functions, physical activity, voice/speech function, BDNF in serum and fMRI (3 T Philips) during performance of motor-cognitive tasks.

**Discussion:**

Since there is currently no cure for PD, findings of neuroplastic brain changes in response to exercise would revolutionize the way we treat PD, and, in turn, provide new hope to patients for a life with better health, greater independence and improved quality of life.

**Trial registration:**

ClincalTrials.gov: NCT03213873, first posted July 11, 2017.

## Background

Parkinson’s disease (PD) is a neurodegenerative disorder with no curative treatment and devastating impact on quality of life of patients and their caregivers, as well as a burden to the health care system [1–3]. It affects the substantia nigra and striatum in the basal ganglia,

which are among the most important structures in the central nervous system playing crucial roles in motor control, learning and high-order cognitive functions [4–6]. In addition, since the basal ganglia have many connections to the rest of the brain, several regions are secondarily affected by the disease. Gait and balance difficulties are prominent early in the disease and are associated with significant disability, decreased quality of life and falls [7]. Cognition, especially executive functions, is often altered in people with PD and characterized by deficits in attention, set shifting, planning, inhibitory control and dual-task (DT) performance [4]. There is also an important connection between gait / balance and cognitive function in people with PD [8] when investigated through the DT paradigm i.e., the performance of a motor task, often gait, concomitant with a cognitive task [9].

Levodopa, considered the gold-standard treatment for PD, has variable effects on cognitive function [4, 10]. Likewise, gait and balance symptoms are just partially alleviated, or even non-responsive, with dopaminergic medication [11–13]. This highlights the importance of non-medical interventions in people with PD. Several systematic reviews and meta-analyses have found various types of exercise to improve a wide range of motor symptoms in PD, including gait ability, balance and strength [14–16]. In addition, promising effects of physical exercise on cognition have been demonstrated in people with PD [17–19]. However, the behavioral effects of exercise on both gait and cognition, as well as dual-task outcomes need further exploration. Above this, it is important to study the underlying neural structures and networks of the brain, especially establishing if, and how, it is altered by exercise [20].

A growing body of research highlights the role of exercise as an essential part of managing PD through potential neuroprotective mechanisms [21]. Multiple structural and physiological mechanisms have been suggested to underlie neuroplastic changes due to exercise in PD, such as increased synaptic strength and a preservation of dopamine neurons [21, 22]. Furthermore, exercise can induce general brain health that might also influence structural and functional properties of the brain [21]. As of yet, studies exploring exercise-induced brain changes in people with PD have been small-scaled and uncontrolled, and have rarely led from pilot studies to full scale randomized controlled trials (RCT). Further, there is missing or underreporting of correlation analysis between improvements in the behaviour and structural / functional improvements in the brain after an exercise intervention, prohibiting firm conclusions to be made.

We previously developed a group training program (the HiBalance program) that combines challenging balance exercises with additional cognitive and motor tasks (dual-tasking) in a progressive manner [23]. The results of a RCT conducted in a hospital research setting showed that participants in the HiBalance program significantly improved their balance and gait, as well as improved cognitive processing during walking compared to a passive control group [19, 24]. Clinical effectiveness was further confirmed after adapting the program from three to two therapist-led sessions weekly and implemented across various clinical settings (in manuscript). As a final evaluative step, we wish to explore whether improvements in gait, balance and cognitive processing could be linked to neuroplastic changes in a double-blinded design with an active control group. The feasibility of the proposed RCT design has been tested and adapted through a pilot study (in manuscript).

Hence, the *purpose* of this study is to determine the effects of a highly challenging balance training (the HiBalance program) on balance performance (primary outcome), gait ability and cognitive function (secondary outcomes) in comparison to an active control group receiving a speech and communication intervention in people with mild-moderate PD. Thereafter, we aim to relate the effects seen in behavior (balance, gait and cognition) to changes in functional connectivity, as well as blood markers of neurobiological plasticity. Our underlying *hypotheses* is 1) that the highly challenging exercise will lead to improved balance performance, gait ability [16, 24] and cognitive function [18, 19] and 2) that the improvements seen in the behavior (balance, gait and cognition) after exercise will be related to altered activity in brain regions that rely on dopaminergic neurotransmission such as the fronto-striatal circuits involved in motor- cognitive control. Finally, we also expect that increased levels of brain-derived neurotrophic factor (BDNF) in serum after the intervention will be related to behavioral changes.

## Methods

### Trial design

The EXPANd (EXercise in PArkinson’s disease and Neuroplasticity) Trial is a randomized controlled trial with a double-blinded design (registered at clinicaltrials.gov NCT03213873). The intervention group will receive the HiBalance program, while the control group receive a speech and communication treatment (HiCommunication) with a similar dose, as well as study setting and deliverance. Reporting of the study follows the SPIRIT (Standard Protocol Items: Recommendations for Interventional Trials) 2013 statement and guidelines.

### Study setting

The assessments will be performed in a university setting with the interventions delivered in a university (academic) hospital setting in Stockholm, Sweden.

### Recruitment and screening

**Fig. 1:**
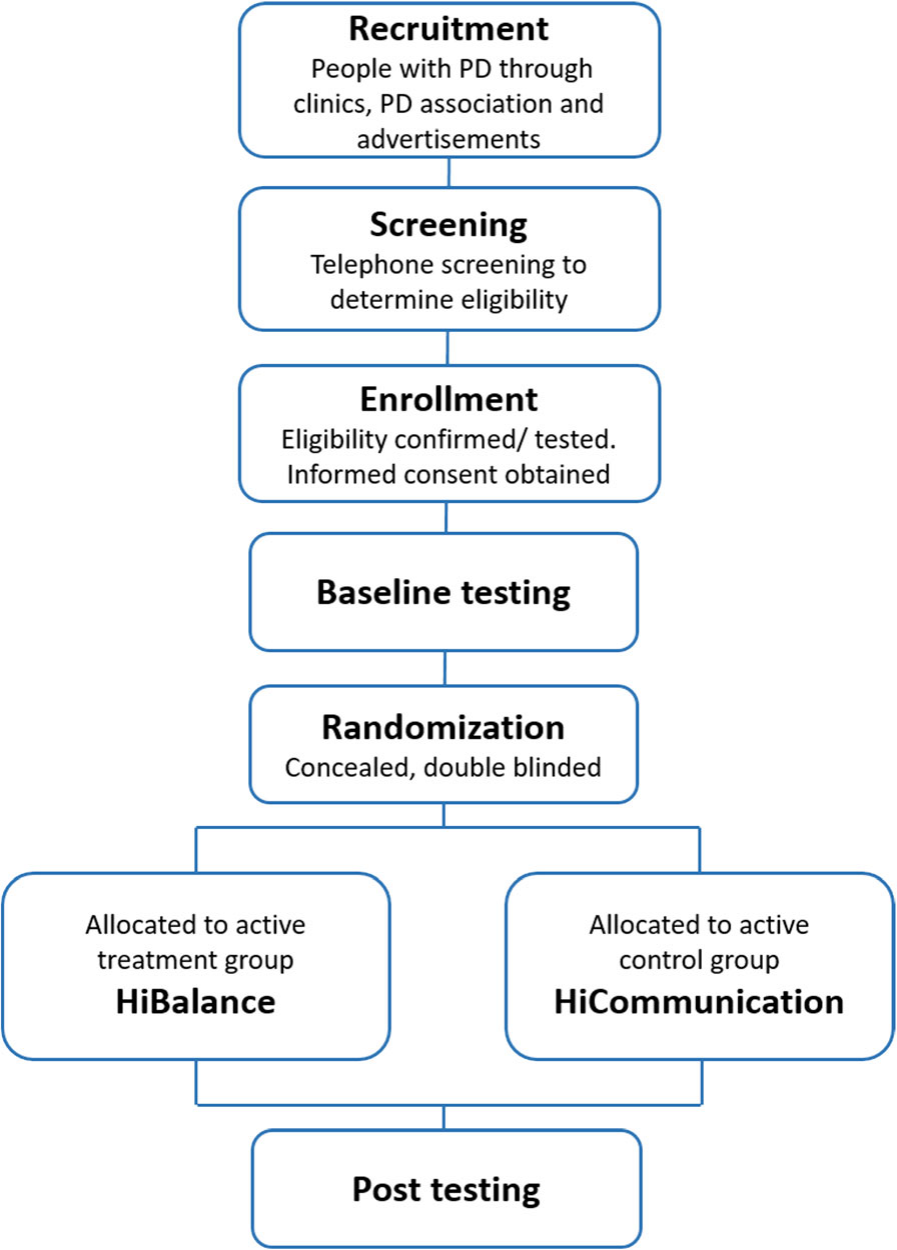
Flowchart of the study

Participants will be recruited through several sources, such as Karolinska University Hospital and other clinics in the vicinity, as well as via announcements in relevant forums, such as the Swedish Parkinson Association and newspapers. Recruitment will be performed in four successive waves, and participants will be randomly assigned (1:1 allocation ratio) to the intervention or the control group in blocks of approximately 25 participants per wave. In each wave, there will be two parallel groups of the intervention, as well as two parallel groups of the active control group. Each group will consist of 6 to 8 participants. Potential participants will initially be screened via telephone to determine eligibility before being asked to visit Karolinska Institutet for a full eligibility exam, see Fig. 1.

### Eligibility criteria

Inclusion and exclusion criteria are compiled in Table 1.

**Table 1:** Study exclusion and inclusion criteria

Inclusion criteria	Exclusion criteria
- Idiopathic PD - Hoehn & Yahr [25] scores 2 to 3 - Stable dose of anti-Parkinson’s medication for approximately 3 weeks - ≥ 60 years of age - Montreal Cognitive Assessment (MoCA) [26] score ≥ 21 - Ambulate indoors without mobility aid - Balance impairments (≤27 on the Mini-BESTest)	- Any other existing disorder that may substantially influence balance performance, voice or speech performance or participation in the interventions - Having participated in an intensive exercise program for balance or speech during the last six months For imaging - Pacemakers, deep brain stimulators or other MRI incompatible implants - Claustrophobia - Unilateral or bilateral blindness - Inability to hear instructions without hearing aid - Severe states of: diplopia, tremor, dyskinesia or dystonia

### Assignment of intervention

Allocation to the intervention or the control group will be blinded to the assessors at all time points, and participants will also be blinded to their group (treatment or control) allocation i.e., the two interventions will be presented as of equal interest in the study. The randomized sequence will be generated on randomization.com by a researcher independent to the project, and participants will receive an opaque envelope with the assigned group following the pre-testing sessions. All information on participants’ group allocation and treating trainers will be kept confidential and non-disclosed from the assessors. At completion of each post-assessment, the assessors will answer a questionnaire to investigate the success of the concealment of the group allocation [27].

### Interventions

#### Intervention group – the HiBalance program

The HiBalance program is based on scientifically well-established principles of exercise training and postural control, as well as current research on exercise in people with PD [23, 24, 28–31]. In brief, the HiBalance program targets four main components of balance control affected in PD (stability limits, anticipatory postural adjustments, sensory integration and motor agility) by principles of motor learning, i.e. specificity, progressive overload and variation, see Table 2. The program also incorporates DT-exercises that gradually integrate cognitive tasks (e.g. counting or remembering items) and motor tasks (e.g. carrying or manipulating an object). To ensure highly challenging exercises, each task is individually adjusted, e.g. by altering the base of support, increasing movement speed/amplitude, restricting vision and varying the grade of multitasking. The difficulty level will be increased in three consecutive blocks, and intermittent presence of reactive postural adjustments will be used as an indicator of the appropriate difficulty level of exercises. Transfer effects and generalization of motor skills to everyday activities will be facilitated by training variation, integrating exercises from the main components with DT exercises. The training will be performed in a clinical setting for 1 h, twice a week for a total of 10 weeks, as a group intervention (6 to 8 participants), and facilitated by two trained physical therapists. In addition, a home exercise program, focusing on aerobic capacity, lower extremity strength and core muscles, will be performed unsupervised in the participants home environment for 1 h once a week [29, 30]. Adherence to the intervention is registered by the trainers.

**Table 2:** Overview of the main components and the progression of the interventions

Intervention	Main Components	Progression (blocks)		
		A week 1–2	B week 3–6	C week 7–10
**HiBalance** (intervention group)	➢ Sensory integration ➢ Anticipatory postural adjustments ➢ Motor agility ➢ Stability limits	Exercises with focus on movement quality, familiarization of the exercises and task-specific motor learning. Single task performance of exercises pertaining to each of the main components.	Increased level of difficulty of the exercises. Variation of the exercises within the components. Introducing cognitive and motor dual tasks to increase the complexity of the exercises.	Complexity increased by task variation and combining exercises from all four main components, and by integrating simultaneous cognitive and motor dual tasks.
**HiCommunication** (control group)	➢ Voice intensity ➢ Articulatory precision ➢ Word retrieval ➢ Memory	Exercises with focus on breathing, phonation and articulation. Establishing increased vocal loudness while maintaining good voice quality	Increased level of difficulty of the exercises. Introducing memory games and associational tasks to increase cognitive load during exercises.	Complexity increased by increasing difficulty of memory games, incorporating more interaction between participants and by adding background noise.

#### Control group – the HiCommunication program

The control group will also receive a group treatment, the HiCommunication program, recently developed at Karolinska Institutet/Karolinska University Hospital. The program targets voice and speech changes associated with PD, specifically aimed at voice intensity, voice quality and articulatory precision [32]. HiCommunication has the same dose as the HiBalance program (1 h, twice a week for 10 weeks) with a 1 h once weekly home exercise program. The program is performed by a speech-language pathologist in groups of 6 to 8 participants. The treatment will aim at increasing vocal loudness and improving articulatory precision, targeting four core areas (voice intensity, articulation, word retrieval and memory). Level of difficulty will be gradually increased in three blocks by progressing from using loud voice and clear speech in short and automatized utterances to using the same technique in more complex sentences and situations, see Table 2. The purpose of gradually increasing the cognitive load while performing the speech training is to enhance transfer of improved speech function to contexts beyond the clinical setting [33]. This intervention will be performed in a sitting position as opposed to the HiBalance intervention that is performed in standing and walking. The home exercise program, to be performed in the home environment for 1 h per week, will focus on relaxation and breathing exercises, as well as voice and speech exercises with additional word retrieval- and memory tasks.

### Adverse events

Adverse events during the intervention period will be reported by the trainers to the research group, using a written form used in other intervention studies [24, 30]. Injurious falls and harmful adverse event will also be reported according to the routines at Karolinska University Hospital.

### Outcomes and procedure

All assessments will be conducted during the ON phase of levodopa medication and at the same time of day at pre- and post-intervention assessments to restrict the influence of medication fluctuations. The assessments will be performed on three different sessions spread across three separate days to minimize fatigue. A general cognitive assessment (MoCA) and assessment of disease severity (Hoehn & Yahr) will be performed to confirm eligibility. Levodopa equivalent daily dose (LED) will be recorded and calculated according to Tomlinson et al. [34]. In addition, height, weight, sex and age, as well as questions regarding prior falls, walking aids and other diseases/disorders will be collected.

First, assessment of gait and balance performance, as well as questionnaires on walking ability, balance confidence, physical activity, anxiety and depression and health related quality of life will be performed. The order of the physical assessments (balance and gait) will be randomised for each participant at both pre- and post-testing to avoid systematic bias due to fatigue. Thereafter, one session will be dedicated to the evaluation of cognitive performance and speech and voice function, and the other to brain imaging. Each session will take 1.5–2 h. In addition, blood will be sampled and habitual physical activity will be assessed at one week before and after the intervention in participants’ home environment using an activity monitor.

#### Primary outcome

The primary outcome of the effect of the HiBalance program will be balance performance assessed with the Mini Balance Evaluation Systems Test (Mini-BESTest), which is a 14 item scale (maximal score 28) assessing four subsystems of balance control (anticipatory postural adjustments, reactive postural control, sensory orientation and dynamic gait) [35, 36]. This scale has been validated for people with PD [37–39].

#### Secondary outcomes

Gait will be assessed under single and DT conditions using an electronic walkway system (GAITRite®, active zone: 8.3 m, CIR Systems, Inc., Havertown, PA, USA), measuring temporal and spatial gait parameters. During DT gait, the Auditory Stroop task will be used [40]. Participants will be presented with the Swedish words for “high” and “low” with congruent and incongruent high and low tones via wireless headphones (Razer™ ManO’War), and asked to respond verbally to the corresponding tone as fast as possible. In order to control for cueing effects, stimuli will be presented with a variable interval (1.5–2 s). Reaction times and number of correct answers will be analysed. Participants will walk back and forth on the GAITRite walkway at self-selected speed during six trials of each condition (single- and DT gait). Acceleration and deceleration distances of three meters on each side of the mat will be provided to ensure steady state walking upon the mat itself [41]. The Auditory Stroop task will also be performed in a sitting position as a comparative single task measure. The order of performance of the single versus DT Auditory Stroop task will be randomized. Importantly, participants will be instructed to pay equal attention to both tasks during DT gait, and standardized practice trials of both gait and the Auditory Stroop task during single- and DT conditions will be performed before the assessments. Dual-task interference (DT performance minus single-task performance) will be calculated for both the cognitive and motor interference according to Rochester et al. [42]. Perceived walking ability will be assessed with the Walk-12G questionnaire, which is a 12 item self-reported rating scale for walking difficulties in everyday life [43].

Physical activity will be assessed with a validated physical activity monitor (Actigraph GT3X+, Manufacturing Technology Inc., Fort Walton Beach, FL, USA) and supplemented with a self-rating scale, the Frändin-Grimby activity scale [44]. The physical activity monitor will be used under free-living conditions, worn around the waist for seven consecutive days, as described in earlier work [45, 46]. Outcomes will be be total physical activity (mean steps per day) and time spent in different intensity levels [47].

Balance confidence during activities of daily living will be assessed with the Swedish version of the Activities-specific balance confidence (ABC) scale [48], where confidence to maintain balance during 16 different real-life situations is rated between 0 (no confidence) and 100% (completely confident).

The Movement Disorders Society Unified PD Rating Scale (MDS-UPDRS) will be used to assess disease related symptoms. The MDS-UPDRS has four parts, I: Non-motor Experiences of Daily Living; II: Motor Experiences of Daily Living; III: Motor Examination; IV: Motor Complications. The scale comprises 65 items altogether [49].

Health related quality of life will be assessed both with a disease specific measurement (the PD Questionnaire, PDQ-39) and with a generic measurement instrument used in a wide range of health conditions and treatments (EQ-5D). EQ-5D consists of five questions/dimensions assessed on a 3 level scale in addition to the evaluation of self-rated health on a vertical visual analogue scale (EQ-VAS) [50]. PDQ-39 is a 39 item questionnaire grouped into eight dimensions of functioning and well-being. The patient reports their difficulties as inflicted by PD during the last month on a 5 point scale regarding daily living, including relationships, social situations and communication [51].

Depression and anxiety will be evaluated with the Hospital Anxiety and Depression Scale (HADS); a self-rating scale consisting of two subscales (anxiety and depression) with eight questions relevant to each subscale [52].

To assess training-related cognitive changes, a neuropsychological battery will be performed, assessing a range of cognitive domains that are commonly affected in people with PD. Executive functions (mainly inhibition, task-set switching, and initiation) will be assessed with the following: 1) Trail Making Test (TMT) trial 4 from Delis-Kaplan Executive Function System (D-KEFS), 2) the Color-Word Interference Test trial 1–4 from D-KEFS, and 3) Verbal Fluency trial 1–3 from D-KEFS [53]. Attention/working-memory (mainly maintenance, manipulation, psychomotor speed, visual search, and sequencing) will be assessed with Digit Span from Wechsler Adult Intelligence Scale - Fourth Edition (WAIS-IV) [54], the TMT trial 1–3, and 5 from D-KEFS. Episodic memory (learning, direct recall, delayed recall, and recognition) will be assessed with Ray Auditory Verbal Learning Test (RAVLT) [55] and Brief Visuospatial Memory Test - Revised (BVMT-R) [56]. Finally, visuospatial functions will be assessed with the Copy condition from BVMT-R.

We will also include speech and voice assessments to evaluate the effects of the control treatment (HiCommunication). The Dysarthria test [57] will be used to assess speech function (including respiration and phonation, oral-motor- and velopharyngeal function and articulation). This test also includes assessment of prosody and intelligibility, as well as a section for self-reported data on perceived speech impairment and communicative participation. In addition, a standardized speech recording, including production of sustained phonation, text reading and production of spontaneous speech will be made in a sound-proofed booth using a head-mounted microphone. Analyses of the speech recording will include voice sound level, mean fundamental frequency and measurements of speech rate, which can also be used for further perceptual analyses.

**Fig. 2:**
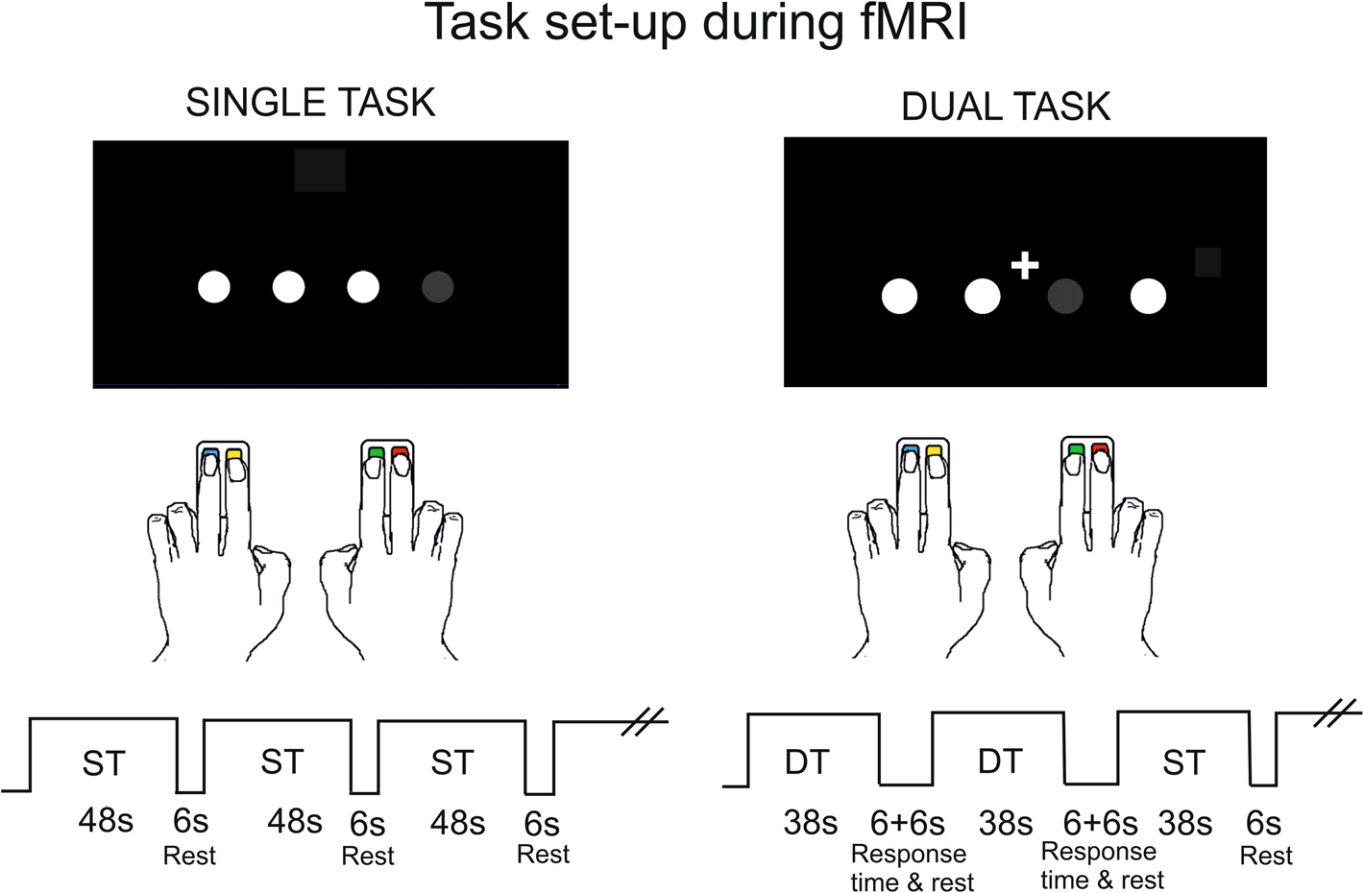
illustrates the experimental set-up of the single tast (ST) and dual-task (DT) during the functional magnetic resonance imaging (fMRI). During both visu-motor tasks four white circles are shown on a black screen with one circle blinking (turning grey) every 1.2 s. The participants will have a 2-button response box in each hand and use their index and middle fingers to respond to the circle turning grey by pressing the corresponding button. During the DT, participants will also perform a counting task, i.e. count how many white plus sign appearing on the screen during the block. After each DT-block, participants will be given four alternatives and respond with the corresponding button how many plus-signs they have counted

Magnetic Resonance Imaging (MRI) will be acquired at one site (MRI Center, Karolinska University Hospital, Huddinge), using a 3 Tesla Philips Ingenia and a 15-channel coil. Subjects will undergo one MRI session before and one after the training period. Each session will include two structural sequences; a T1 scan (6 min) and a T2 scan (5 min). It will also include two task-evoked fMRI sequences; a visuo-motor task consisting of visually informed finger movements and a DT designed as the visuo-motor task but with the addition of a simple counting task. In the visuo-motor task, four white circles will be shown on a black screen with one circle blinking (turning grey) every 1.2 s. The participants will have a 2-button response box in each hand and use their index and middle fingers to respond to the circle turning grey by pressing the corresponding button (see Fig. 2). Both tasks will have a block-design with each block interleaved by a 6-s rest. Each block of the visuo-motor task will consist of 40 visual stimuli (i.e., circles turning grey) and the DT will consist of blocks of 32 visual stimuli. In the DT, the visuo-motor task is combined with a simple counting task in five of the blocks. These five blocks are followed by an extra six seconds where the participant is required to give an answer to the counting task. To make the participant aware of the approaching dual task, these blocks will also be preceded by all four circles shortly turning green.

All participants will be trained outside the scanner on both the visuo-motor and DT with a similar set-up of buttons as used inside the scanner. MR-compatible glasses will be provided when necessary, and earplugs and headphones will always be used to protect against noise. Subjects will be instructed to lie as still as possible and head padding will be used to help subjects keep their heads still.

Brain-derived neurotrophic factor (BDNF) will be assessed from blood serum, before and after the exercise interventions, and analyzed with ELISA commercial analyzing kit. Venous blood will be collected into sampling tubes at a hospital sampling central. The samples will be centrifuged, aliquoted and stored at − 80 °C until analysis.

To enable comparison between the two groups with regard to motivation, expectancy of symptom relieve and credibility of the intervention, a 10-item questionnaire inspired by Devilly & Borkovec [58] will be filled in by the participants three weeks into the intervention. After the intervention period, both groups will also answer an anonymous questionnaire regarding the adverse events during the training period, their perception of the therapist-led exercise, and the home exercise.

### Ethics and data handling

The trial has been approved by the Regional Ethical Review Board in Stockholm 2016/1264–31/4, 2017/1258–32 and 2017/2445–32. Participants will receive written and oral information about the study and all assessments, as well as provide written informed consent before the start of the assessments. Only authorized researchers will have access to the data. Data will be pseudonymized and stored in paper and digital format in accordance with regulations regarding public authority archives and the General Data Protection Regulation.

### Sample size

We calculated the power based on 2000 bootstrap samples from data generated on parameters obtained from pilot data. The pilot data consisted of 12 individuals with PD, 5 from one group, and 7 from the other. By testing the interaction term of a random-intercept model with group (binary), time (binary), and their interaction as covariates, we found that a sample size of 40 individuals with PD per intervention would give a power of 82% to detect a between-group difference of two points in mean of the total score of the Mini-BESTest at post-testing. Based upon our previous studies [19, 24], this sample size would also be sufficient to adequately power some of the secondary outcomes (gait velocity, step length and dual-tasking). We also account for expected imaging related exclusions due to technical problems or head/body motions. Taken together, to ensure statistical power, we would need to include 50 in each intervention group.

### Statistical analysis

Descriptive statistics will be used to describe the groups at baseline. We will employ a random effects general linear model analysis to handle unbalanced/missing data. Group (HiBalance and HiCommunication) and time (pre- and post-intervention) will be factors in the model that determine the effects of the interventions. Group-by-time interaction, main effect of group and time analyses will be evaluated. To compare the magnitude of gains, effect size will be calculated.

For the imaging data, we will primarily investigate changes in functional connectivity with a focus on the fronto-striatal network, but with the addition of a whole-brain analysis. To link behavioral changes in balance performance to changes in the brain, we will perform correlational analysis using the Spearman linear correlations within the respective groups. Sub-group analysis in relation to disease stage or baseline cognitive performance might be performed to check for heterogeneity of the effects. In case of skewed distribution of the data, corresponding non-parametric statistics will be used to assess main effects of the intervention. The a-priori alpha level for analyses of 0.05 with adjustments for multiple testing will be used when appropriate.

## Discussion

This is the first study to compare the HiBalance program with a double-blinded design and an active control treatment incorporating a similar dose, design and deliverance, which takes into consideration the social aspects of a group treatment and the engagement of health care personnel. Using structural and functional MRI together with levels of BDNF before and after training will allow us to explore neuroplasticity changes due to the exercise interventions from two perspectives. The ultimate goal of this study is to link behavioural effects of a highly challenging balance exercise program to changes in functional connectivity in the fronto-striatal network, as well as blood markers of neurobiological plasticity in people with mild-moderate PD.

The overall trial design has been found to be feasible and acceptable in a pilot study (in manuscript). From the pilot study, we have further strengthened the design by using a double-blinded design, as well as optimized the MRI protocol to avoid diplopia and daytime somnolence, amended the exclusion criteria (e.g. added criteria for blindness, diplopia, tremor, dyskinesia or dystonia), streamlined the blood sampling process and analysis, as well as added a participant expectancy and credibility questionnaire.

There are some limitations to the proposed study, although the total dose of ant-Parkinson medication will be monitored from a longitudinal perspective, separating the potential effects of the interventions from those of levodopa medication will be limited. On the other hand, as mentioned in the introduction, the effects of Levodopa on balance, gait and cognition are ambiguous, in that it may worsen gait and balance [13].

This study design will also allow us to explore many other questions regarding motor and cognitive performance and corresponding brain activity, as well as explore the effects of a new speech and communication treatment for people with mild-moderate PD. The knowledge gained from this trial could accelerate the understanding of the link between motor and cognitive function and how motor-cognitive exercise changes the function of the brain, as well as the behaviour. Such results can revolutionize the way we treat people with PD and possibly also other neurodegenerative disorders, as well as provide new hope to patients for a longer life with better health, greater independence and improved quality of life.

### Trial status

Recruitment of participants began in December 2017 and data collection started in January 2018 and is expected to go on until December 2019.

## Data Availability

The datasets generated during and/or analysed during the current study are not publicly available due to Swedish and EU personal data legislation but are available from the corresponding author on reasonable request. Any sharing of data will be regulated via a data transfer and user agreement with the recipient.

## Abbreviations

ABC: Activities-specific balance confidence
BDNF: brain-derived neurotrophic factor
BVMT-R: Brief Visuospatial Memory Test - Revised
BVMT-R: Brief Visuospatial Memory Test – Revised
D-KEFS: Delis-Kaplan Executive Function System
DT: dual-task
fMRI: Functional magnetic resonance imaging
HADS: Hospital Anxiety and Depression Scale
LED: Levodopa equivalent daily dose
MDS-UPDRS: Movement disorder society version of the unified Parkinson disease rating scale
Mini-BESTest: Mini Balance Evaluation Systems Test
MoCA: Montreal Cognitive Assessment
MRI: Magnetic resonance imaging
PD: Parkinson’s disease
PDQ-39: Parkinson’s disease questionnaire
RAVLT: Ray Auditory Verbal Learning Test
RCT: randomized controlled trial
SPIRIT: Standard Protocol Items: Recommendations for Interventional Trials
SRTT: serial reaction time task
TMT: Trail Making Test
WAIS-IV: Wechsler Adult Intelligence Scale - Fourth Edition.
